# Intramuscular fat is present in cervical multifidus but not soleus in patients with chronic whiplash associated disorders

**DOI:** 10.1371/journal.pone.0197438

**Published:** 2018-05-24

**Authors:** Ashley Pedler, Katie McMahon, Graham Galloway, Gail Durbridge, Michele Sterling

**Affiliations:** 1 Recover Injury Research Centre, NHMRC Centre of Research Excellence in Recovery Following Road Traffic Injury, The University of Queensland, Brisbane, Australia; 2 Menzies Health Institute Queensland, Griffith University, Gold Coast, Australia; 3 Centre for Advanced Imaging, University of Queensland, Brisbane, Australia; 4 Translational Research Institute, Brisbane, Queensland, Australia; Rush University Medical Center, UNITED STATES

## Abstract

The presence of intramuscular fat (IMF) in the cervical spine muscles of patients with whiplash associated disorders (WAD) has been consistently found. The mechanisms underlying IMF are not clear but preliminary evidence implicates a relationship with stress system responses. We hypothesised that if systemic stress system responses do play a role then IMF would be present in muscles remote to the cervical spine. We aimed to investigate if IMF are present in muscle tissue remote (soleus) to the cervical spine in people with chronic WAD. A secondary aim was to investigate associations between IMF and posttraumatic stress symptom levels. Forty-three people with chronic WAD (25 female) and 16 asymptomatic control participants (11 female) participated. Measures of pain, disability and posttraumatic stress symptoms were collected from the WAD participants. Both groups underwent MRI measures of IMF in cervical multifidus and the right soleus muscle. There was significantly greater IMF in cervical multifidus in patients with WAD and moderate/severe disability compared to controls (p = 0.009). There was no difference in multifidus IMF between the mild and moderate/severe disability WAD groups (p = 0.64), or the control and mild WAD groups (p = 0.21). IMF in the right soleus was not different between the groups (p = 0.47). In the WAD group, we found no correlation between PDS symptoms and cervical multifidus IMF or between PDS symptoms and soleus IMF. Global differences in IMF are not a feature of chronic WAD, with differences in IMF limited to the cervical spine musculature. While the mechanisms for the development of IMF in the cervical spine following whiplash injury remain unclear, our data indicate that local factors more likely contribute to these differences.

## Introduction

Clearly identifiable pathological causes for ongoing pain in whiplash associated disorders (WAD) remain elusive. Imaging studies have generally failed to find a higher prevalence of cervical spine pathology in people post whiplash injury than in asymptomatic controls [1–3]. The exception to this are recent magnetic resonance imaging (MRI) studies that have identified the presence of higher levels of intramuscular fat (IMF) in people with WAD when compared to asymptomatic controls and people with non-traumatic neck pain [4–6]. A longitudinal investigation found that these fatty deposits develop between 2 and 12 weeks post whiplash injury and are significantly greater in people with poor functional recovery at 6 month follow up [7]. The significance of IMF in terms of patient outcomes and treatment are not clear. Whilst preliminary evidence suggests the presence of IMF is associated with poor recovery [7], prognostic capacity has not been investigated in an adequately powered cohort study that also includes known prognostic indicators. It may be that IMF develops secondary to other factors such as loss of movement or disuse. Nevertheless an understanding of mechanisms underlying IMF may contribute to further understanding of aetiological processes of WAD.

Whilst mechanisms underlying the development of IMF in cervical spine muscles have not been established, possible mechanisms have been proposed. These include inflammation, denervation, disuse, altered activation of the sympathetic nervous system and stress system dysregulation [7] and some of these have undergone preliminary investigation. In patients with WAD, no strong relationships were found between inflammatory cytokine levels and cervical IMF [8] but posttraumatic stress symptom levels have been shown to mediate the relationship between pain in acute injury stage and later 6 month development of cervical IMF [9]. Stress related symptoms and elevated inflammatory cytokine levels are features of WAD[10–12], with some suggesting WAD is a systemic condition rather than one localised to the neck[13]. This latter finding suggests that responses associated with stress symptoms maybe be a contributing factor to the development of IMF. Activation of the stress system has wide ranging physiological sequelae including cardiovascular, metabolic, inflammatory and immune system responses [14, 15]. While beneficial in the short term, prolonged stress system activation can have deleterious effects. Prolonged increased sympathetic outflow, hypercortisolemia, and pro-inflammatory cytokines associated with stress system activation can have deleterious effects on skeletal muscle [16, 17]. Patients with WAD and elevated levels of distress also show increased cortisol levels [18] and decreased heart rate variability [19].

If systemic stress system responses play a role in the development of IMF following whiplash injury, it would seem logical that that muscles away from the cervical spine may also be affected. Further, should widespread muscular differences be present in people with WAD this may have significant functional and general health consequences and may influence treatment approaches in this population. The aim of this study was to investigate if differences in IMF are present in muscle tissue remote to the cervical spine, in this case right soleus, in people with chronic WAD. Soleus muscle was investigated in an attempt to compare the cervical multifidus to a geographically and functionally remote muscle with a largely postural role. A secondary aim was to further investigate relationships between IMF and posttraumatic stress symptom levels.

## Materials and methods

### Study design

A cross-sectional study comparing two participant groups (chronic WAD and healthy controls).

### Participants

Participants with chronic WAD and asymptomatic control participants were recruited through general advertisement in South East Queensland, Australia. Participants with chronic WAD were also recruited from a previously collected database of patients held by the researchers. Participants with WAD Grade II (neck pain and physical signs of movement loss, neck tenderness) were included provided they were currently experiencing neck pain as the result of a motor vehicle crash (MVC) which occurred more than 3 months ago but not more than 10 years ago and provided that they rated their neck pain over the past week as moderate or greater (modified item seven of Short Form 36 survey [20]–the question asked about neck pain as opposed to bodily pain in the original item). Inclusion criteria for both participant groups were; aged 18–65 years, no history of mental illness, head injury, diabetes or other medical condition which may influence results, no injury or pain in the right leg requiring treatment in the past 12 months and no contraindications for MRI examination. Patients with WAD III (neurological loss) and WAD IV (fracture or dislocation) were excluded [21].

In addition, the asymptomatic control participants were included provided they had no history of whiplash injury, were pain free and had no history of neck pain over the preceding 12 months. Asymptomatic control participants were matched to the WAD participants with respect to age grouping and gender.

Participants were provided with a small reimbursement for travel costs to attend for assessment. All participants provided written informed consent prior to participation. Ethical approval to conduct the study was gained from the Human Ethics committee of The University of Queensland.

### Procedure

Following initial contact, potential participants were screened for eligibility and MRI safety via telephone. Eligible participants were provided with information regarding the study and were invited to participate. Those who agreed to participate attended the university to undergo MRI examination where they provided written informed consent then completed written questionnaires. Participants were then screened for MRI contraindications by the attending radiographer before undergoing MRI examination of the cervical spine and right leg. Total scanning time was approximately 45 minutes. The order of scanning was randomised between participants.

### Outcome measures

#### Demographics

Injury-related and demographic data were recorded including age, gender, employment status, medication use, previous treatment, 24 hour and 7 day average pain intensity (10cm VAS), accident details, compensation status and medical history.

#### Neck Disability Index

Self-reported pain related disability was assessed with the Neck Disability Index (NDI). This scale consists of 10 items relating to pain and daily function rated on a 6 point scale ranging from no pain or disability to severe pain and disability. Each item is scored from 0–5 and individual item scores are summed and multiplied by 2 to provide a score out of a possible 100 with higher scores indicating higher disability. This scale has been widely used in research in people with WAD [22, 23] and is valid [24] and reliable (Cronbach α 0.74 to 0.93) [24, 25].

#### Posttraumatic Diagnostic Scale

The severity of posttraumatic stress symptoms were assessed using the total symptom severity subscale of the Posttraumatic Diagnostic Scale (PDS). This subscale consists of 17 items describing symptoms of posttraumatic stress which are rated on a 4 point scale based on the frequency with which the symptom has been experienced in the previous month with responses ranging from ‘Not at all or only one time’ (0) to ‘5 or more times a week/almost always’ (3). Scores from each item are summed to produce a total score out of a maximum of 51 with higher scores representing higher symptom severity. This scale has been used previously in samples people with WAD and has been shown to be valid [26] and reliable (Cronbach α 0.95) [27].

#### MRI scanning procedure

All images were acquired on a Siemens 3T Trio MRI scanner (Trio, Siemens, Erlangen, Germany). A 3D-gradient echo chemical shift based 2-point Dixon method was used to acquire images of the cervical spine and right calf musculature. A 12-channel head coil and the posterior aspect of the dedicated neck coil were used to acquire cervical spine images and a dedicated knee coil was used to collect images of the calf musculature. A T1 Volume Interpolated Gradient Echo (VIBE) using Dixon fat-water separation sequence was performed with the following parameters; TR = 5.6ms, TE1 = 2.45ms, TE2 = 3.675ms, axial slices of 4mm, flip angle of 9 degrees, FOV = 260mm and a voxel of 0.8*0.8*4mm. This procedure collects data at echo times when water and fat are in-phase and out of phase producing two images—a fat only image and a water only image. IMF of the region of interest (ROI) is calculated from mean pixel intensity of the fat only and water only images using the formula:

IMF(%)=Fat/(Fat+Water)×100

**Fig 1:**
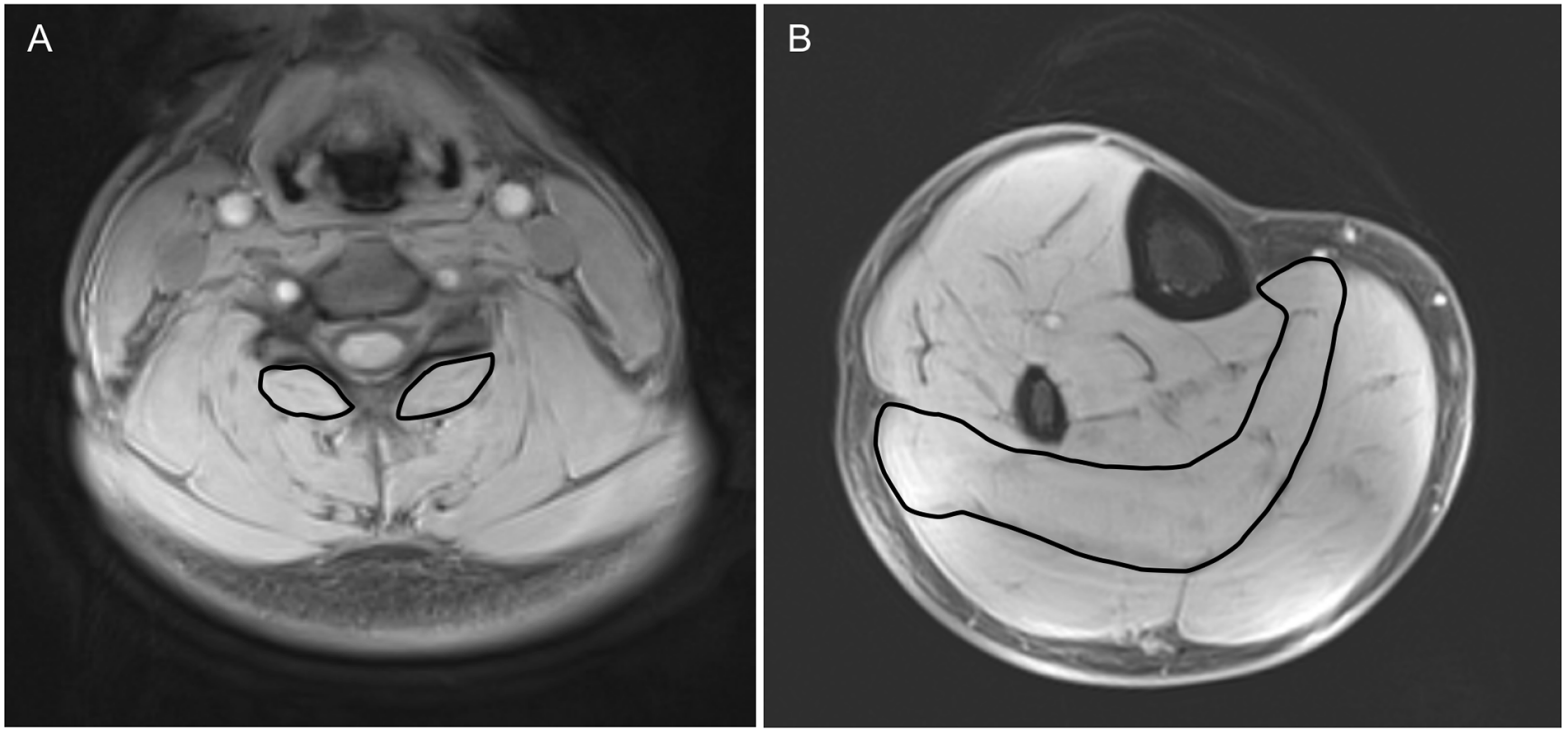
Regions of interest of A) segmental multifidi muscles at C6 and B) soleus of the water only images from a set of T1 weighted VIBE Dixon sequences.

In the present study IMF was calculated through manually tracing ROIs of the bilateral cervical multifidus from the C3 spinal level to the C7 spinal level and the right soleus at the level of the tibial tuberosity to the lowest visible level of the muscle belly on separate fat only and water only images. Fig 1 illustrates the ROIs for the multifidus muscle at C3 segmental level and soleus in a healthy control participant. For cervical multifidus, ROIs were traced at 3 points for each cervical level using axial images. For the C3 level, the 3 slices corresponding to the most caudad portion of the vertebral body were used. For all remaining levels, slices corresponding to the most cephalad, midpoint and most caudad portion of the vertebral body were used. Due to a high level of artefact observed at the C7 level in several cervical spine imaging sequences, data for the C7 cervical level was excluded from analysis for all subjects. Strong intra- and inter- rater reliability has been demonstrated for this methodology [28]. A volume for the right soleus was generated through tracing an ROI encompassing the muscle at every 5 slices in the sequence. ROIs for the remaining slices were created through automatic interpolation within Analyze 12.0 software (AnalyzeDirect Inc., USA). The interpolated ROIs were then manually checked and adjusted where required to create the final volume. IMF for each pair of fat and water ROIs was calculated using custom software developed in Python (http://www.python.org) [29]. A mean IMF value was calculated for the cervical multifidus across each side using bilateral IMF values from each of 3 slices at each cervical level. In order to account for between subject variations in lower leg length resulting in different proportions of the soleus being imaged, mean IMF of the middle 50% of the soleus volume was used in all analyses.

We chose to investigate the soleus muscle in an attempt to compare the cervical multifidus to a geographically and functionally remote muscle with a largely postural role. We also chose to include only the cervical multifidus as this muscle has been shown to be have the greatest level of IMF in patients with WAD[6, 7, 30, 31].

#### Data analysis

In order to determine if differences in IMF was present in the soleus and multifidus of participants with chronic WAD, the chronic WAD group was sub-grouped based on NDI scores into those reporting mild (NDI < 30%) and those reporting moderate to severe (NDI ≥ 30%) neck disability[32]. Previous work has shown that higher IMF in the cervical spine develops in those reporting higher levels of disability [7, 9]. Between-group differences in IMF for the cervical multifidus and the soleus were examined through a multivariable ANCOVA. Age, gender and body mass index (BMI) were included as covariates in the analysis. Planned comparisons were made between groups using Bonferroni adjustments for multiple comparisons. In order to determine any independent association of posttraumatic stress symptoms with IMF in the chronic WAD group, multivariable linear regressions were performed. The independent variables were IMF in soleus and multifidus, with correlate variables of age, gender, time since injury, BMI, average pain and PDS score used in both regressions. Significance levels were set to p < 0.05 for all analyses. All statistical analyses were performed using IBM SPSS Statistics 22 (IBM, USA).

A priori sample size calculations were calculated and based on previous data comparing IMF between people with chronic WAD and healthy controls using the same MRI measure [28]. A sample size of 15 participants per group was calculated as sufficient to detect between group differences in IMF of 9% with a standard deviation of 6% at an alpha level of 1% with power of 90%.

## Results

### Participants

Forty-three people with chronic WAD (25 female, mean age = 39.4 +/- 11.1 years) and 16 asymptomatic control participants (11 female, mean age = 38.6 +/- 12.6 years) agreed to participate in this study. Group characteristics are reported in Table 1. Twenty-two of the WAD participants (51.2%) reported that they had lodged a compensation claim with 8 (18.6%) reporting that their claim had been settled. Sixteen (37.2%) participants had engaged the services of a solicitor and 23 (53.5%) were taking at least 1 medication for their neck pain at the time of assessment. Eighteen (41.9%) were taking analgesics, 13 (30.2%) muscle relaxants and 10 (23.3%) were taking antidepressants or antianxiety medications.

**Table 1:** Study group demographics.

	Healthy Controls	Mild NDI	Moderate/Severe NDI
**Age (yrs)**	39.3 (12.6)	39.3 (11.0)	39.5 (11.5)
**Gender (% F)**	66.7	50.0	69.6
**BMI**	22.8 (3.4)	25.9 (4.6)	25.5 (4.3)
**24hr average pain**		3.8 (2.0)	5.2 (1.6)
**NDI (%)**		19.6 (5.2)	46.1 (13.7)
**PDS**		5.2 (5.0)	16.6 (12.3)
**Symptom duration (weeks)**	**-**	274.1 (164.0)	181.3 (133.5)

BMI = body mass index, NDI = Neck Disability Index, PDS = Posttraumatic Stress Diagnostic Scale.

### Between group comparisons

#### Cervical multifidus and soleus IMF

**Fig 2:**
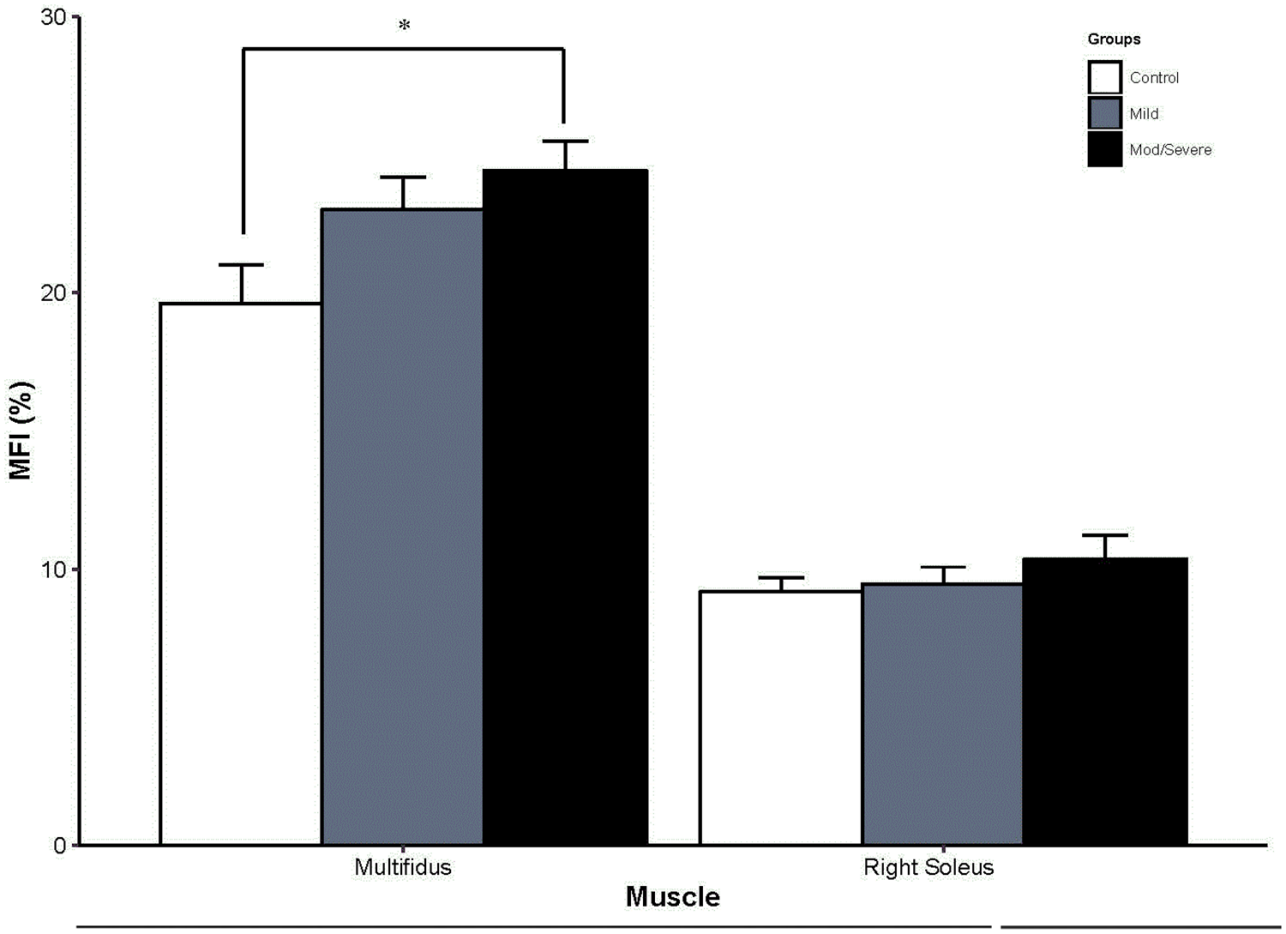
IMF of cervical multifidus and soleus by study group. * indicates statistically significant between group differences at *p* < 0.05.

There was a significant main effect for group on multifidus IMF (F[2,49] = 6.3, p = 0.004). Age had a statistically significant effect on multifidus IMF (F[1, 49] = 9.4, p = 0.004) but BMI did not (F[1, 49] = 0.5, p = 0.48). Pairwise comparisons showed a significantly greater multifidus IMF in the moderate/severe WAD group compared to the control group (p = 0.001). There was no difference in multifidus IMF between the mild and moderate/severe WAD groups (p = 0.08), or the control and mild WAD groups (p = 0.06, Fig 2).

#### Soleus IMF

Both age (F[1, 53] = 14.2, p < 0.001) and BMI (F[1,53] = 29, p = 0.05) had significant effects on soleus IMF. There was no statistically significant main effect of group on soleus IMF (F[2, 53] = 0.50, p = 0.60, (Fig 2).

#### Regression analyses

Age was the only significant correlate of multifidus IMF (β = 0.196, p = 0.004). Gender (β = 1.2, p = 0.45), time since injury (β = -0.003, p = 0.58), BMI (β = -0.043, p = 0.81), average pain (β = 0.399, p = 0.31) and PDS score (β = 0.025, p = 0.72) were not significant correlates of multifidus IMF. The overall model fit was R^2^ = 0.26.

Age was also the only significant correlate of soleus IMF (β = 0.139, p = 0.005). Gender (β = -0.26, p = 0.82), time since injury (β = -0.005, p = 0.15), BMI (β = -0.26, p = 0.054), average pain (β = -0.38, p = 0.21) and PDS score (β = 0.04, p = 0.42) were not significant correlates of soleus IMF. The overall model fit was R^2^ = 0.37.

## Discussion

Previous work has identified elevated levels of IMF in the cervical spine musculature of people with chronic WAD [4–6, 31]. Longitudinal investigation has shown that cervical muscle IMF develops only in patients with later poor recovery, defined as moderate or greater neck pain related disability at 6 months post injury [8, 9]. Posttraumatic stress symptom levels were a significant mediator of the relationship between initial pain and later levels of IMF [9] implicationg stress system responses in the development of IMF. If this were the case, then we hypothesised that IMF may also occur in muscles away from the cervical spine. We found no significant differences in IMF in the soleus between people with chronic WAD and a demographically similar asymptomatic control group, despite between-group differences in IMF at the cervical multifidus. Our data indicate that it is more likely that local mechanisms contribute to the development of IMF in the cervical spine musculature post whiplash injury.

Despite growing evidence demonstrating alterations in muscle morphology of the cervical musculature in people with WAD, the mechanisms underlying these changes are not clear. There are plausible physiological mechanisms available to explain associations between IMF and stress system responses, including effect of increased sympathetic outflow resulting in vasoconstriction of small blood vessels supplying skeletal muscles [17]. We have previously shown a relationship between stress symptoms and IMF, however the results of the present study do not support our previous findings. We also found no relationship between posttraumatic stress symptoms and muscle morphology of soleus. Our findings indicate that there is no link between stress responses and IMF in local and remote muscles of people with WAD.

Previous findings could be explained by posttraumatic stress symptoms being a proxy for pain related disability. IMF develops predominantly in patients reporting higher disability. The development of both disability and posttraumatic stress symptoms occur in close parallel and similar factors predict both outcomes [11, 33]. This could suggest that stress related responses play no role in the development of IMF. Alternative causal influences may be inflammatory processes, although preliminary studies have not found support for this proposal [34] or simply that whiplash patients reporting higher disability move their head and neck less or in altered ways [35] and this in turn leads to muscle degeneration.

A recent observational case study reported higher IMF in the bilateral lower limbs in 3 people with chronic WAD in comparison to 1 person who had recovered from a whiplash injury [36]. Methodological differences may account for the divergence of our results. We considered only the soleus while Elliott et al. reported total IMF for all lower limb muscles and did not report differences between specific muscles. We confined our data to the soleus in an attempt to compare the cervical multifidus to a geographically and functionally remote muscle with a largely postural role. Our study was also powered *apriori* and we were able to control for the effect of age and BMI in our statistical analyses. Both these factors showed associations with soleus IMF. However, we cannot discount that our findings may be different if additional lower limb muscles were included.

We have not accounted for the effect of physical activity on IMF in the present study. It is possible that differences in physical activity between groups may lead to differences in muscle morphology. The inclusion of a measure of physical activity, either self-report or through accelerometry would increase the certainty with which between-group differences in muscle morphology could be interpreted in the future. In addition we did not blind the assessor responsible for MRI analysis as to whether participants were in the WAD or healthy control groups. However the scoring of questionnaires occurred following calculation of IMF ensuring the assessor was blinded to levels of posttraumatic symptoms during MRI analysis. Given the potential for bias these results should be interpreted with caution until these results can be replicated.

Our data show that higher IMF is present in the cervical multifidus but not the soleus in people with chronic WAD. We found no association between posttraumatic stress symptoms and IMF in cervical multifidus or the soleus. These findings suggest that global differences in IMF are not a feature of chronic WAD, with higher IMF limited to the cervical spine musculature. While the mechanisms for the development of IMF in the cervical spine following whiplash injury remain unclear, our data indicate that local factors may be more likely.

## Supporting information

S1 Study Data (XLSX)
